# Atlantoaxial Subluxation in a 10-Year-Old Girl With Down Syndrome: A Case Report

**DOI:** 10.7759/cureus.43955

**Published:** 2023-08-23

**Authors:** Sumaiah Alfhmi, Nevein Sejeeni, Khawlah Alharbi, Rahaf Alharbi, Baraah Malayoo

**Affiliations:** 1 Pediatric Medicine, Maternity and Children Hospital, Makkah, SAU; 2 Faculty of Medicine, Umm Al-Qura University, Makkah, SAU

**Keywords:** ataxia, dislocation, instability, atlantoaxial subluxation, down syndrome

## Abstract

Down syndrome is the most common inherited chromosomal disorder caused by trisomy 21. Atlantoaxial instability (AAI) is more common in children with Down syndrome, resulting from ligament laxity and odontoid dysplasia. We report the case of a 10-year-old girl with Down syndrome submental. She came to the ER with a history of abnormal gait for one week and was admitted with a case of ataxia for investigations. Moreover, we discovered that she had atlantoaxial subluxation, which was treated surgically.

## Introduction

Down syndrome is the most common inherited chromosomal disorder caused by trisomy 21, with a prevalence estimate of one in 700 live births [1]. Atlantoaxial dislocation is defined by a loss of stability between the atlas and the axis (C1-C2), which results in a loss of standard articulation. Traumatic, inflammatory, idiopathic, or congenital abnormalities can cause the atlantoaxial joints to lose stable articulation [2]. Atlantoaxial instability (AAI) is more common in children with Down syndrome than in normal children, resulting from ligament laxity and odontoid dysplasia. However, 14.6% to 22.2% of individuals with Down syndrome are affected by AAI [3]. Spitzer first reported it in 1961 [4]. Various measurements and imaging studies can be used to diagnose cervical spine instability. The most common parameter used to diagnose atlantoaxial instability is the ADI, defined as the distance between the posterior surface of the anterior arch of C1 and the anterior surface of the dens. According to these parameters, 10% to 30% of patients with Down syndrome have radiographic atlantoaxial instability [5-9]. Symptomatic C1-to-C2 instability affects only about 1% of patients with Down syndrome. Myelopathy or other neurological findings rarely correlate with radiographic abnormalities. There are insufficient long-term studies on the natural history of craniovertebral instability in patients with Down syndrome, and there is controversy regarding the optimal treatment [1]. We report a case of a 10-year-old girl known as a case of Down syndrome submental. She came to the ER with a history of abnormal gait for one week.

## Case presentation

**Figure 1:**
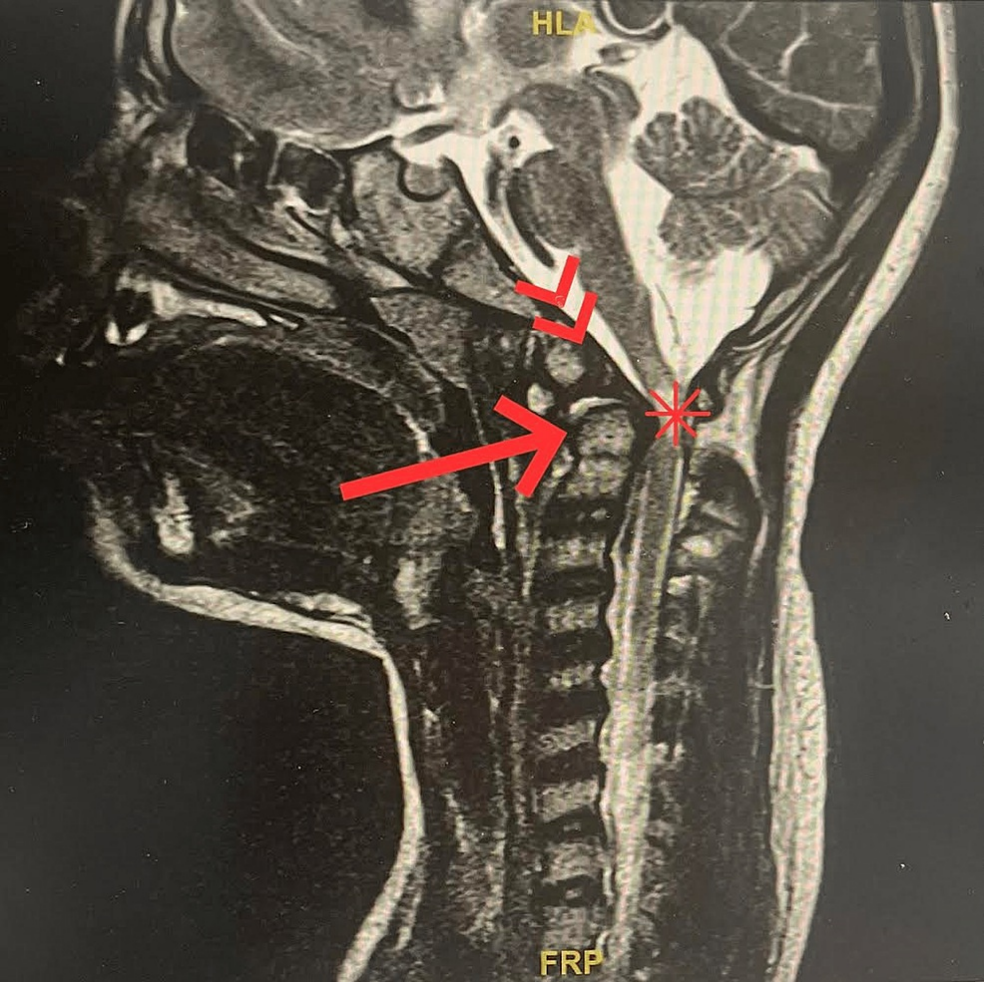
Brain MRI Normal variant Os odontoid (double arrow) superimposed with a retroverted odontoid process (arrow) results in focal compression of the upper cervical cord and subsequent chronic myelopathy (asterisk).

A 10-year-old girl with the typical features of Down syndrome was admitted to our hospital with an abnormal gait for one week, which her mother had noticed. She was delivered as a sister to seven healthy siblings and admitted to the NICU for one week for poor feeding. She also has a known case of bronchial asthma (on salbutamol as needed) and has had previous gastroenteritis hospitalizations. She was vaccinated up to her age. On this admission, the ataxic gait was the only symptom in presentation; otherwise, there was no history of fever, headache, other abnormal movements, change of consciousness, light sensitivity, or any focal neurological symptoms. No history of trauma that might lead to her abnormal gait was mentioned. However, on a neurological examination, horizontal nystagmus was found, and there was increased muscle tone and deep tendon reflexes in the lower extremities with ataxic gait; the upper extremities were normal in power, tone, and tone reflexes. A neurological consultation was done, and they ordered a brain CT; it was unremarkable, and there was no clinical correlation. Brain MRI confirmed the diagnosis of atlantoaxial subluxation with subsequent compressive myelopathy at the C1 level (Figure 1). The patient was referred to a specialized neurosurgery center; surgery was performed, where fixation was followed by extensive physiotherapy and occupational therapy. The patient fully recovered after six weeks.

## Discussion

Atlantoaxial dislocation is instability between the atlas and axis (C1-C2), which results in abnormal articulation [2]. In addition, patients with Down syndrome have also been reported to have low bone mineral density, transverse ligament laxity, low muscle tone, and excessive joint flexibility compared to the general population. However, at this time, the researchers could not explain the cause of the AAOD anomaly in many Down syndrome patients [10-15]. Most studies on this topic were published in the 1980s and 1990s [16]. In children, dislocation is considered present if the Atlanta-odontoid interval is more significant than 4.5 mm [17]. After finding an Atlanta-odontoid interval of 8 mm in a 10-year-old asymptomatic girl with Down's syndrome, a study was done to examine the cervical spine radiographs of 18 people. They found that the Atlanta-odontoid interval was more significant than 5 mm in four cases when measured at the inferior aspect of the anterior Atlanta-odontoid joint [18]. Another study discovered that if the Atlanto-odontoid interval was between 4.5 and 6.0 mm, the patients were free of neurological symptoms; however, if the distance was more significant than 7.0 mm, all patients had neurological symptoms. [19]. Moreover, our patient has an abnormally increased atlantoaxial distance measuring 5.5 mm, and significant posterior subluxation of the dens is seen, with the cervical cord being compressed between the dens and the posterior arch of the atlas. Some authors recognize the importance of analyzing neurological symptoms in order to properly diagnose potential spinal cord compression while emphasizing that some people with DS, due to cognitive disorders and intellectual disability, may be unable to recognize the symptoms and, as a result, may not report them to their relatives [16]. A study presented intriguing findings, analyzing the relationship between the occurrence of AAI and neurological symptoms and finding no correlation whatsoever [9]. The physical examination should identify a head tilt or torticollis and eliminate evidence of limb weakness, increased tone, and reflexes, indicating cervical spinal cord compression. Before elective general anesthesia, every patient with Down syndrome should have lateral radiographs of the cervical spine in flexion and extension to rule out asymptomatic atlantoaxial instability. Asymptomatic individuals who do not have radiographs should try to avoid any movements that forcefully or excessively flex, extend, or rotate the neck. Furthermore, any individual with Down syndrome with evidence of spinal cord compression, either in the history or on examination, should be referred to a neurosurgical center immediately to consider spinal fusion [20].

## Conclusions

Atlantoaxial instability is a common occurrence in patients with Down syndrome. Because of the association between Down’s syndrome and atlantoaxial dislocation, screening cervical radiographs to assess for atlantoaxial instability in Down’s syndrome patients should be done initially. Surgical correction is indicated if radiographic results or neurological symptoms suggestive of spinal cord injury are present.

## References

[1] Song D, Maher CO (2007). Spinal disorders associated with skeletal dysplasias and syndromes. Neurosurg Clin N Am.

[2] Yang SY, Boniello AJ, Poorman CE, Chang AL, Wang S, Passias PG (2014). A review of the diagnosis and treatment of atlantoaxial dislocations. Global Spine J.

[3] Takeoka Y, Kakutani K, Miyamoto H (2021). Complications of posterior fusion for atlantoaxial instability in children with Down syndrome. Neurospine.

[4] Ali FE, Al-Bustan MA, Al-Busairi WA, Al-Mulla FA, Esbaita EY (2006). Cervical spine abnormalities associated with Down syndrome. Int Orthop.

[5] Menezes AH, Ryken TC (1992). Craniovertebral abnormalities in Down's syndrome. Pediatr Neurosurg.

[6] Brockmeyer D (1999). Down syndrome and craniovertebral instability. Topic review and treatment recommendations. Pediatr Neurosurg.

[7] Pueschel SM, Scola FH (1987). Atlantoaxial instability in individuals with Down syndrome: epidemiologic, radiographic, and clinical studies. Pediatrics.

[8] Pizzutillo PD, Herman MJ (2005). Cervical spine issues in Down syndrome. J Pediatr Orthop.

[9] Roy M, Baxter M, Roy A (1990). Atlantoaxial instability in Down syndrome--guidelines for screening and detection. J R Soc Med.

[10] Carfì A, Liperoti R, Fusco D (2017). Bone mineral density in adults with Down syndrome. Osteoporos Int.

[11] Center J, Beange H, McElduff A (1998). People with mental retardation have an increased prevalence of osteoporosis: a population study. American Journal on Mental Retardation.

[12] González-Agüero A, Vicente-Rodríguez G, Gómez-Cabello A, Casajús JA (2013). Cortical and trabecular bone at the radius and tibia in male and female adolescents with Down syndrome: a peripheral quantitative computed tomography (pQCT) study. Osteoporos Int.

[13] Matute-Llorente A, González-Agüero A, Gómez-Cabello A, Vicente-Rodríguez G, Casajús JA (2013). Decreased levels of physical activity in adolescents with down syndrome are related with low bone mineral density: a cross-sectional study. BMC Endocr Disord.

[14] McKelvey KD, Fowler TW, Akel NS, Kelsay JA, Gaddy D, Wenger GR, Suva LJ (2013). Low bone turnover and low bone density in a cohort of adults with Down syndrome. Osteoporos Int.

[15] Sepulveda D, Allison DB, Gomez JE, Kreibich K, Brown RA, Pierson RN Jr, Heymsfield SB (1995 Low spinal and pelvic bone mineral density among individuals with Down syndrome. Am J Ment Retard.

[16] Myśliwiec A, Posłuszny A, Saulicz E (2015). Atlanto-axial instability in people with Down’s syndrome and its impact on the ability to perform sports activities-a review. J Hum Kinet.

[17] Whaley WJ, Gray WD (1980). Atlantoaxial dislocation and Down's syndrome. Can Med Assoc J.

[18] TI J, MA W (1965). Dislocation of the atlas in mongolism: preliminary report. Radiology.

[19] Pueschel SM, Herndon JH, Gelch MM, Senft KE, Scola FH, Goldberg MJ (1984). Symptomatic atlantoaxial subluxation in persons with Down syndrome. Journal of Pediatric Orthopaedics.

[20] Powell JF, Woodcock T, Luscombe FE (1990). Atlanto-axial subluxation in Down's syndrome. Anaesthesia.

